# Dynamic intraligamentary stabilization: novel technique for preserving the ruptured ACL

**DOI:** 10.1007/s00167-014-2949-x

**Published:** 2014-03-21

**Authors:** S. Eggli, H. Kohlhof, M. Zumstein, P. Henle, M. Hartel, D. S. Evangelopoulos, H. Bonel, S. Kohl

**Affiliations:** 1 Sonnenhof Orthopaedic Clinic, Swiss Leading Hospitals, Buchserstrasse 30, 3006 Bern, Switzerland; 2 Department of Orthopaedic Surgery, Inselspital, University of Bern, Freiburgstrasse, 3010 Bern, Switzerland; 3 Department of Trauma Surgery, University Medical Center Hamburg-Eppendorf, Martinistraße 52, 20246 Hamburg, Germany; 4 3rd Department of Orthopaedic Surgery, University of Athens, Christovassili Street, Neo Psychikon, 15451 Athens, Greece; 5 Department of Radiology, Inselspital, University of Bern, Freiburgstrasse, 3010 Bern, Switzerland

**Keywords:** Anterior cruciate ligament, Dynamic intraligamentary stabilization, Arthroscopic surgery, Sports injury, Knee injury

## Abstract

**Purpose:**

Replacement of the torn anterior cruciate ligament (ACL) with a transplant is today`s gold standard. A new technique for preserving and healing the torn ACL is presented. Hypothesis: a dynamic intraligamentary stabilization (DIS) that provides continuous postinjury stability of the knee and ACL in combination with biological improvement of the healing environment [leucocyte- and platelet-rich fibrin (L-PRF) and microfracturing] should enable biomechanically stable ACL self-healing.

**Methods:**

Ten sportive patients were treated by DIS employing an internal stabilizer to keep the unstable knee in a posterior translation, combined with microfracturing and platelet-rich fibrin induction at the rupture site to promote self-healing. Postoperative clinical [Tegner, Lysholm, International Knee Documentation Committee (IKDC), visual analogue scale patient satisfaction score] and radiological evaluation, as well as assessment of knee laxity was performed at 6 weeks, 3, 6, 12, and 24 months.

**Results:**

One patient had a re-rupture 5 months postoperative and was hence excluded from further follow-ups. The other nine patients presented the following outcomes at 24 months: median Lysholm score of 100; IKDC score of 98 (97–100); median Tegner score of 6 (range 9–5); anterior translation difference of 1.4 mm (−1 to 3 mm); median satisfaction score of 9.8 (9–10). MRI showed scarring and continuity of the ligament in all patients.

**Conclusions:**

DIS combined with microfracturing and L-PRF resulted in stable clinical and radiological healing of the torn ACL in all but one patient of this first series. They attained normal knee scores, reported excellent satisfaction and could return to their previous levels of sporting activity.

**Level of evidence:**

Case series with no comparison group, Level IV.

## Introduction

Rupture of the anterior cruciate ligament (ACL) is the most common injury of the knee requiring surgical treatment [18]. While a conservative treatment approach leads to satisfactory results in a population that places low demand on the knee joint [31, 41], persisting instability prevents patients from participating in activities that require high levels of joint pivoting, such as soccer and skiing.

Today’s gold standard in ACL repair was developed by Brückner in 1966 and uses the middle third of the patellar ligament as transplant to restore knee joint stability [10]. Although arthroscopic techniques have improved tremendously since then, and current ACL reconstruction techniques are an excellent option for restoring sagittal plane stability of the knee, the clinical results with tendon grafts are still under discussion. Despite numerous publications reporting good-to-excellent results, the meta-analysis of Biau et al. [8] revealed that only 40 % of patients achieve full recovery independent of surgical technique. One explanation could be that removal of the native ACL tissue containing sensory nerve fibres causes the ligament to lose its function within the joint’s ‘proprioceptive envelope’ [3, 23], thus impairing muscular stabilization of the knee. Based on this theory, the authors started to investigate a strategy for preserving the torn ACL in 2007.

The main challenge is posed by the torn ligament’s poor healing capacity. This can be partly explained by biological factors such as cell deficiencies, alterations in cellular metabolism, the hostile environment of the synovial fluid [12, 42], and the lack of blood supply [2, 26]. Moreover, the postinjury instability does not allow the torn ligament to heal.

While recent studies support the potential of biological self-healing for the ruptured ACL [34, 35, 38], the persisting postinjury translation in the antero-posterior plane separates the ligament stumps by 5–10 mm and prevents possible self-healing and formation of stable scar tissue [1, 16, 43]. To address this problem, a new technique was developed, dynamic intraligamentary stabilization (DIS), hypothesizing that continuous posttraumatic stabilization of the knee can enable mechanically stable ACL healing. Encouraged by the success of DIS in a sheep model [27], the technique was applied in a series of 10 physically active individuals with a torn ACL.

## Materials and methods

Inclusion criteria were an ACL rupture not older than 14 days, patient age <45 years, no previous surgery on the injured knee, and regular participation in sports requiring pivoting of the knee joint. Ten consecutive patients (eight males, two females) met the inclusion criteria and underwent surgery between August 2009 and February 2010. Median age was 25.4 years (range 19–41 years); the right/left knee ratio was 7/3. The median accident-surgery interval was 9.9 days (range 2–13 days). The rupture was located in the middle third of the ligament in seven patients and in the proximal third in three patients. Eight patients showed additional meniscal lesions, which were surgically treated in six patients.

## Surgical technique

**Fig. 1:**
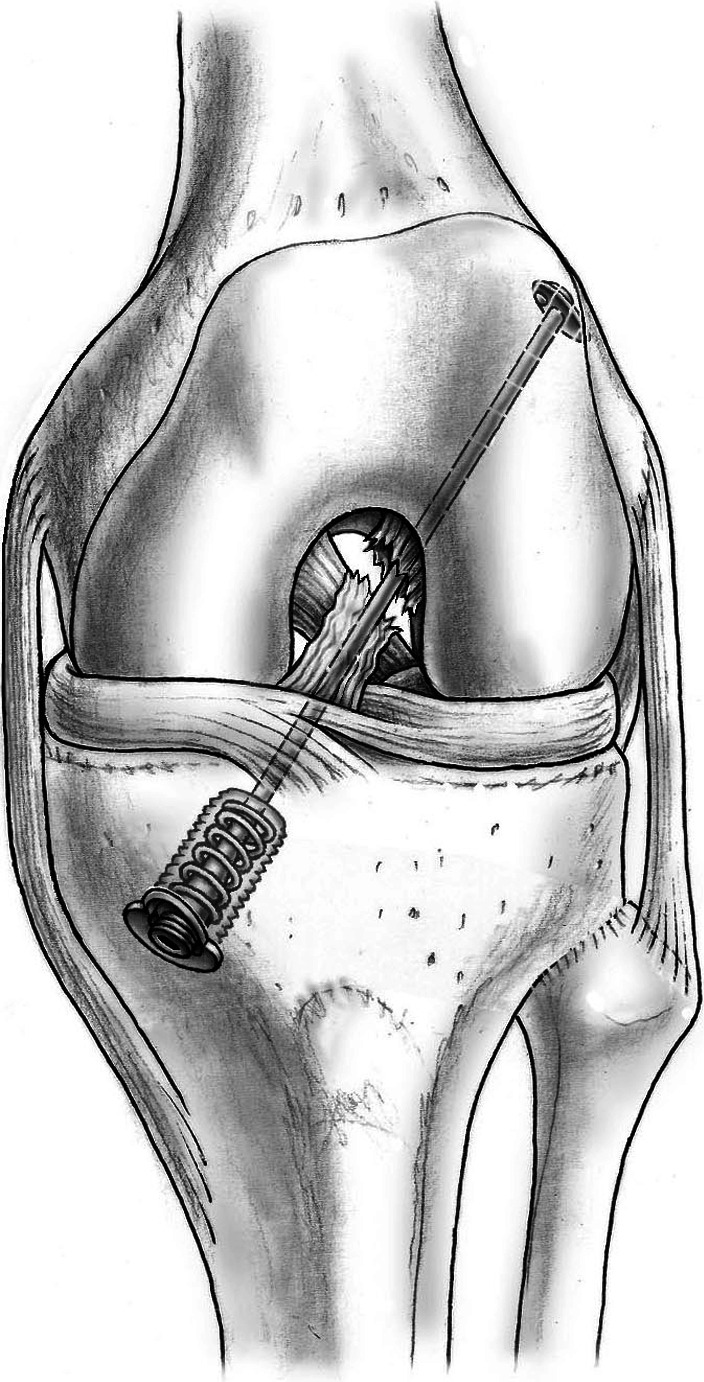
Dynamic screw-spring mechanism pushes the tibia into a posterior translation at every degree of flexion

Each patient was placed in a supine position with the knee positioned in a static knee holder with a tourniquet inflated to 350 mm Hg. An antero-lateral portal was used for the camera and an antero-medial portal for the instruments. The infrapatellar area was freed from the hypertrophic portions of Hoffa’s fat pad. Removal of too much tissue, especially from the inferior part of the fat pad, was avoided to preserve nutritional arteries to the ACL passing through this area [2]. The tibial footprint of the ACL was marked using an intra-articular guide, and a wire was passed through the tibia to this point. A 10.5-mm threaded sleeve (Mathys Ltd, Bettlach, Switzerland) was then inserted into the tibia. A suture passer was inserted through the screw into the distal ACL stump, and a preliminary thread was passed through the ligament. The femoral footprint was identified using a guide from the antero-medial portal, and a wire was passed at 120 degrees of flexion to the lateral aspect of the femur. The wire was passed through the skin, and the definitive polyethylene wire was inserted from the antero-lateral femoral position to the antero-medial aspect of the tibia. The wire was stabilized at the femoral position with a flip anchor. A metallic spring was inserted in the screw, and the polyethylene wire was fixed with a cover at the end of the screw at a tension of 80 N. The DIS technique holds the knee in a fixed posterior translation at every degree of flexion, ensuring that the two ligament stumps are kept as close to each other as possible at all times (Fig. 1). The surgery was then completed by microfracturing of the femoral footprint and induction of a leucocyte- and platelet-rich fibrin (L-PRF) clot at the rupture site.

Postoperative treatment consisted of the patient spending 3 days in a fixed position with the knee fully extended. The leg was then loaded with 15 kg of weight for 3 weeks and mobilized without any flexion limitations. Beginning at 4 weeks postoperative, the leg was loaded with full body weight and reinforcement training of the quadriceps and hamstrings was initiated using closed chain knee exercises. Intensive proprioceptive training was initiated with a trampoline and balancing exercises on an unstable board. Running was allowed after 6 weeks and pivoting sports after 3 months. Competitive soccer and skiing were allowed after 5 months.

## Clinical evaluation

All patients were evaluated according to a prospective protocol, 6 weeks, 3, 6, 12, and 24 months after surgery. The following instruments were used for outcomes assessment at each follow-up: Tegner score, Lysholm score, the International Knee Documentation Committee (IKDC) score, and visual analogue scale (VAS) for assessment of patient satisfaction (0 = completely dissatisfied, 10 = completely satisfied). The preoperative scores were assessed retrospectively using the same questionnaires. Knee laxity was assessed by measuring anterior translation at 30 degrees flexion using a Rolimeter™ (Aircast, Neubeuern, Germany) and comparing it with the contralateral knee. All patients were informed that their treatment would involve a completely new technique, to which they gave voluntary written informed consent (Cantonal Ethics Committee of Berne, Switzerland: Ref.-Nr. KEK-BE: 048/09, ISRCTN 89368687).

## Radiological evaluation

Conventional radiographic evaluation was performed immediately after surgery and at the 6-week and 12-month follow-ups. MRI investigations were conducted 6 weeks, 3, 6, and 12 months after the intervention using an advanced 3 Tesla Scanner (Magnetom Verio; Siemens, Erlangen, Germany) with a dedicated 15-channel knee coil.

All examinations were reported in random order by a specialized radiologist with a more than 15-years experience in musculoskeletal radiology. The radiologist was blinded to all clinical information except for the surgical repair of the ACL. First, image quality was assessed using a 5-point scale (5 = optimal image quality, 4 = very good image quality, 3 = diagnostic image quality, 2 = anatomic structures only identified, 1 = anatomic structures not seen). Next, the MRIs were examined for intactness of the ligament by an independent evaluator applying the following criteria:

### Morphology and continuity

The ligament tear was rated in three grades similar to those used by Kühne et al. [28] after ACL repair, grade 3 representing a non-delineated ligament, grade 2 a wavy but continuous ligament contour, and grade 1 a continuous ligament.

### Signal intensity

The 4-level grading system for the PCL, as proposed by Howell et al., was adapted to analyse variations in the signal intensity parameters of the graft [21]. In grade I, a homogeneous, low-intensity signal was observed within the entire graft segment. In grade II, at least 50 % of the ‘normal’ ligament signal was observed. In grade III, the graft segment depicted less than 50 % of the normal ligament signal. In grade IV, there was a diffuse increase in signal intensity with abnormal ligament strands.

### Statistical analysis

To express the variability and distribution of the underlying data, the median values of outcome scores and their range were calculated and reported. No inferential statistics were used in this exploratory descriptive study (*n* = 10).

## Results

At 5 months after surgery, patient number 6 (a 24-year-old male sports student) suffered from a re-rupture after sustaining a direct rotation trauma playing soccer. Until then, the patient had been pain-free with a Lysholm score of 97 and a Tegner score of 5 at the 3 month follow-up. 6 weeks after the second trauma his completely ruptured ACL was replaced by a bone-tendon-bone (BTB) graft.

The remaining nine patients were all monitored according to the clinical follow-up protocol and had a complete set of MRI investigations. The Lysholm score was 100 before injury, 99 (97–100) after 3 months, and 100 after 24 months. The IKDC score reached 98 (97–100) after 24 months, and with six points the group`s median Tegner activity score remained the same as before the accident. After 3 months, the anterior translation difference was 0.5 mm (−3 to 3) compared with the contralateral side and 1.4 mm (−1 to 3) after 24 months. Before surgery, it was 4.9 (range 3–7 mm, SD 1.2 mm). The anterior stop was hard in three patients and semi-hard in six patients. Median patient satisfaction was 9.5 (8–10) after 3 months and 9.8 (9–10) after 24 months (Table 1).

**Table 1:** Clinical scores at follow-up examinations (median values and range)

Scores/tests	Before injury	3 Months	6 Months	12 Months	24 Months
Lysholm	100	99.1 (97–100)	99.8 (99–100)	99.8 (98–100)	100
IKDC	100	92.1 (87–96)	94 (90–98)	97.8 (97–100)	98 (97–100)
Tegner	6 (4–9)	5 (4–6)	5 (4–8)	6 (5–9)	6 (5–9)
Δ Lachmann (mm)		0.5 (−3–3)	1.0 (−3–3)	1.2 (−2–3)	1.4 (−1–3)
Pat. satisfaction VAS		9.5 (8–10)	9.6 (9–10)	9.8 (9–10)	9.8 (9–10)

### MRI

Imaging quality was rated as optimal (score 5) in nine patients and very good (score 4) in one patient at 6 weeks. Metal artefacts were detected at the site of the implanted spring mechanism. Metal artefacts obscured 18 % of the distal ACL (range 12–23 %) but did not alter the morphology of the proximal and middle third of the ligament.

Continuity was rated as grade A, or ‘well defined’, in all nine patients at all times. Patient number 6 was also rated grade A before his re-rupture. All repairs were well defined, and ligament continuity was fully restored.

**Fig. 2:**
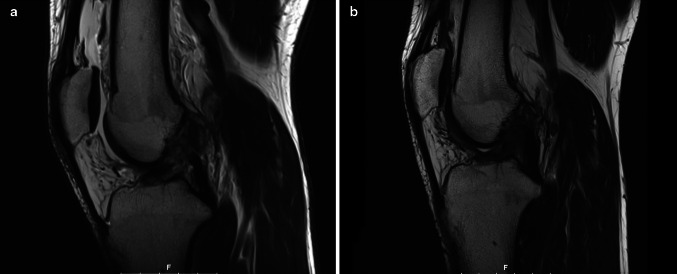
Lateral MRI showing the torn ACL immediately after injury and at 12 months follow-up (after removal of screw)

Applying the Howell grading system, MRI signals were rated I in 2 of the 10 patients after 6 weeks and II in the remaining 8 patients. After 3 months and for all later assessments, the signals were rated I for all patients except patient number 6 after the second rupture of his ligament (Fig. 2).

## Discussion

The most important finding of the present study was that a dynamic intraligamentary stabilization of the knee with a freshly ruptured ACL in combination with biological improvement of the healing environment can lead to a biomechanically stable ACL with good functional scores and high patient satisfaction.

ACL rupture is a devastating knee injury that is associated with a significant risk of developing osteoarthritis [15]. Controversially, this risk is reported to be even higher in patients undergoing surgical stabilization of the ACL [11, 25, 29]. These problems may be attributable to loss of proprioception after complete removal of the ACL [5, 31, 37] as well as to insufficient restoration of the three-dimensional stability of the knee [6, 36, 39]. It was therefore hypothesized that conservation of the native ACL tissue is necessary to preserve proprioception and restore the individual three-dimensional anatomy of the ligament.

The paradigm that a torn ACL has insufficient healing capacity and must be replaced by a tendon graft remains prevalent [14, 20]. Opposing this paradigm, a series of publications have indicated that under certain circumstances the injured ACL can produce a stable scar. Träger et al. [43] reported a better clinical outcome and improved stability with suturing the ACL and augmenting the tissue with polydioxanone than with ACL replacement. Steadman et al. [40] described a method known as the ‘healing response’ wherein placement of undifferentiated stem cells into the rupture zone induced stable healing of the torn ACL in an athletically active, skeletally immature patient. In 2002, Fujimoto et al. [17] published the results of 31 ACL ruptures in patients with low athletic demands treated conservatively for 2–3 months with an extension block soft brace without anterior stabilization: 23 knees (74 %) were stable with a continuous ACL on MRI at final follow-up. Boldrini et al. [19] reported stable healing in 26 athletes with an incomplete ACL tear using primary sutures in combination with bone marrow stimulation.

The literature to date indicates that stability and biology are the two main determinants for ACL healing. Every tissue demands a certain level of stability to heal [24], but since a knee with an injured ACL shows a significant increase in antero-posterior translation of the tibio-femoral joint [13], normal knee movements result in a constant disconnection of the two ACL stumps, creating an unstable healing environment. The published method of knee bracing in a constant posterior translation for 3 months has already produced a remarkable number of healed ligaments, but it is not widely accepted because of the discomfort it causes [17, 22]. The authors have developed a new technique called dynamic intraligamentary stabilization (DIS) that restores the intrinsic stability of the knee with minimal discomfort for the patient. The DIS device employs an internal screw-spring mechanism that acts as a dynamic internal fixator which pushes the knee into a maximum posterior translation in any degree of flexion. The spring is also functional when the intraligamentary thread is not placed in an isometric position. This technique has already been shown to provide sufficient mechanical stability to enable biomechanically stable healing of the ACL in a sheep model [27].

Recent studies have demonstrated that introduction of a collagen-platelet composite into a transected ACL can significantly increase its healing capacity [30, 32, 33]. Murray et al. [32] used a collagen-platelet composite to bridge the wound site and reported healing with recovery of over 50 % of the initial ligament strength after 4 weeks. Zumstein et al. [44, 45] have demonstrated that solid scaffolds can be used for long-term (up to 28 days) delivery of growth factors; in particular, L-PRF can be used as a regenerative in situ tissue engineering method during treatment of ACL injuries. This technique was adapted in combination with microfracturing according to Steadman to optimize the biological healing capacity of the ACL.

The MRI investigations at 3, 6, and 12 months showed a continuous increase in scar tissue as represented by fibres of low signal intensity. In addition, clear remodelling of the ligament was detectable at 6 months, and the independent evaluator judged the continuity of the ligaments as restored. Furthermore, the dynamic aspect of the repair resulted in a straight appearance of the fibre bundles without the wavelike morphology observed with other surgical techniques.

Clinically, all patients exhibited practically normal knee function after 1 year with a Lysholm score of almost 100 and an IKDC score of 98. These results are strikingly improved in comparison with the outcome after conventional ACL repair with a tendon graft. However, the reported ceiling effects of the Lysholm score need to be considered in this series of highly motivated and active patients [9]. A better discriminating outcome instrument may be needed for these types of patients in the future. In their meta-analysis, Biau et al. [8] reported that only about 40 % of patients made a full recovery after ACL reconstruction, with only 33 % having a normal IKDC score after a semitendinosus transplant and 41 % having a normal IKDC after a BTB (ligamentum patellae) transplant. Thus, more than 60 % of patients (708 of 1,125 for the two reconstruction groups) did not fully recover (final overall IKDC score class A) after reconstruction.

The present authors attribute the excellent clinical results of their new technique to the restored stability of the knee on one hand, but even more to the preservation of the ACL tissue, which allows for the restoration of a physiological proprioception. Barret points out that ligament tests and knee scores correlate poorly with patient satisfaction scores, but proprioception is a major factor in measuring the overall outcome of ACL reconstruction [4]. Patients in the present study reported a satisfaction of 9.8 on VAS after 1 year, indicating that healing of the ACL tissue may restore not only the three-dimensional stability of the knee but also the physiological proprioceptive envelope. Casteleyn states that the real benchmark of success in the treatment of ACL ruptures has to be the avoidance of treatment morbidity, secondary surgery, and osteoarthritis [11]. In the meta-analysis of Biau et al. [7], 13–22 % of the patients complained of persisting knee pain, mainly associated with donor-site morbidity. The DIS technique requires no additional ligament extraction, thus minimizing the surgical trauma.

There are limitations to this study. The sample size is small, there is no control group, and the follow-up is not long enough to address posttraumatic arthritis, which results in an evidence level of only IV.

The authors conclude that this study could revitalize the discussion of preserving the torn ligament. It could be an additional option between the conservative treatment and the replacement of the ACL. Further studies need to be carried out to proof this concept.

## Conclusion

Dynamic intraligamentary stabilization in combination with L-PRF and microfracturing of the notch can lead to stable clinical and radiological healing of the torn ACL after 1 year. Patients exhibited normal knee function reported excellent satisfaction and were able to return to their previous levels of sporting activity. The present findings support the discussion of a new paradigm in ACL treatment based on preservation and self-healing of the torn ligament.
